# Identification of QTL for Target Leaf Spot resistance in *Sorghum bicolor* and investigation of relationships between disease resistance and variation in the MAMP response

**DOI:** 10.1038/s41598-019-54802-x

**Published:** 2019-12-04

**Authors:** Jennifer Kimball, Yaya Cui, Dongqin Chen, Pat Brown, William L. Rooney, Gary Stacey, Peter J. Balint-Kurti

**Affiliations:** 1 Department of Agronomy and Plant Genetics, University of Minnesota, St. Paul, MN 55108 USA; 2 Divisions of Plant Science and Biochemistry, C. S. Bond Life Science Center, University of Missouri, Columbia, MO 65211 USA; 3 Department of Plant Sciences, University of California Davis, Davis, CA 95616 USA; 4 Department of Soil and Crop Sciences, Texas A&M University, College Station, TX 77843 USA; 5 Dept of Entomology and Plant Pathology, NC State University, Raleigh, NC 27695 USA; 6 Plant Science Research Unit, USDA-ARS, Raleigh, NC 27695 USA

**Keywords:** Plant immunity, Plant stress responses

## Abstract

Target leaf spot (TLS) of sorghum, a foliar disease caused by the necrotrophic fungus *Bipolaris cookei (*also known as *Bipolaris sorghicola)*, can affect grain yield in sorghum by causing premature drying of leaves and defoliation. Two sorghum recombinant inbred line (RIL) populations, BTx623/BTx642 and BTx623/SC155-14E, were assessed for TLS resistance in replicated trials. Using least square mean trait data, four TLS resistance QTL were identified, two in each population. Of these, three were previously unidentified while a major QTL on chromosome 5 in the BTx623/BTx642 RIL population corresponded to the previously identified TLS resistance gene ds1. A set of sorghum lines were assessed for production of reactive oxygen species induced by treatment with the microbe-associated molecular pattern (MAMP) flg22 (a derivative of flagellin). Flg22-induced ROS production varied between lines in a consistent fashion. One QTL associated with variation in the flg22 response was detected in the RIL populations. No evidence was found to link variation in the MAMP response to variation in TLS resistance

## Introduction

Target leaf spot (TLS) of sorghum, a foliar disease caused by the necrotrophic fungus *Bipolaris cookei (*also known as *Bipolaris sorghicola)*, can affect grain yield in sorghum by causing premature drying of leaves and defoliation. TLS is a significant disease of sorghum in the southeastern US and the lower Mississippi river valley^1^ and has been reported worldwide including India^2^, Japan^3^, and the United States^4^.

Few studies have been published on the genetics of TLS resistance in sorghum. A major recessive TLS resistance gene, *ds1*, on chromosome 5 appears to be caused by a loss-of-function or loss-of-expression allele of a gene encoding a putative plasma membrane receptor with leucine-rich repeat and serine-threonine kinase domains^3^. Mohan *et al*.^5^ identified quantitative trait loci (QTL) associated with resistance to TLS on chromosomes 3 and 6. The chromosome 6 QTL was associated with resistance to two other diseases, zonate leaf spot and drechslera leaf blight, in addition to TLS^6^. To our knowledge, these are the only three loci that to have been associated with TLS resistance.

Plants recognize pathogens using a suite of receptors located on the plasma membrane and in the cytoplasm. Plasma membrane-bound receptors, known as pattern recognition receptors (PRRs) generally recognize microbe-associated molecular patterns (MAMPs, also known as pathogen-associated molecular patterns or PAMPs), highly conserved molecules generally found in large classes of microbes^7^. The archetypal MAMP is flg22, a 22-amino acid epitope derived from bacterial flagellin^8,9^. Other well-studied MAMPs include epitopes of chitin, lipopolysaccharides (LPSs), peptidoglycans (PGNs), and translation elongation factor Tu (EF-Tu)^10^. MAMP recognition by PRRs leads to a relatively low-level defense response termed the MAMP response or the MAMP-triggered immunity (MTI) response. In cases in which pathogens are specifically adapted to the host plant, pathogen derived proteins known as effectors are often introduced into the cytoplasm or apoplast. Some effectors can suppress the MTI response while others play various roles in enhancing pathogenesis^11^. Cytoplasmic resistance proteins (R-proteins) recognize effectors either indirectly or via direct binding, leading to a relatively strong defense response termed the effector-triggered immunity (ETI) response. The ETI and MTI responses are qualitatively similar though ETI is stronger. Both can include phenomena such as cell wall reinforcement by callose deposition, changes in ion flux across the plasma membrane, changes in phytohormone concentrations, induction or repression of plant defense-related genes, and production of reactive oxygen species (ROS) and nitric oxide (NO)^12^. ETI often (though not always) includes a so-called hypersensitive response (HR) – a rapid, localized cell death at the point of pathogen penetration^13^.

In some necrotrophic systems, necrotrophic effectors, also known as host-specific toxins, cause the tissue necrosis specifically in plants carrying dominant susceptibility (S-) genes^14^. Most of the S-genes that have been identified encode proteins similar to R-genes or, in some cases, PRRs^15–18^. It seems that in these cases the pathogen may induce the host to trigger HR which will result in dead cells on which the necrotrophic pathogen can grow. It is not clear how many necrotrophic pathogens use this strategy beyond the four or five systems that have been characterized already^19^ but new cases are being discovered regularly. There are several reasons to suspect that the TLS resistance gene *ds1* may be an example of an S-gene that is triggered by a host-specific toxin; susceptibility is dominant, the structure of *ds1* resembles a PRR and the causal pathogen, *B. cookei*, is a necrotroph which is closely related to several pathogens that produce host-specific toxins^20^. The host-specific toxin, if it exists, has not been identified in this case.

Quantitative trait loci (QTL) controlling the MTI response have been documented in a number of plant species including *Arabidopsis thaliana*^21,22^, maize^23^, soybean^24^, and tomato^25^. At the same time, it has become increasingly apparent that quantitation of the MTI response is complex. Correlations in populations between responses to different MAMPs has been reported to be low or non-existent in some cases^21,25,26^ though Zhang *et al*.^23^ reported a strong correlation between responses to flg22 and chitin in a maize mapping population. Moreover, the MTI response can be quantified in various ways using various assays including measuring ROS or NO production; MAP kinase phosphorylation, mRNA levels of specific MAMP-induced genes, lignin and cell wall-bound phenols, seedling growth inhibition and MAMP-induced resistance to pathogens^26–28^. Relative rankings of lines can vary substantially depending on the assay used^23,27^. In other words, quantification of the MTI response is not straightforward and conclusions may vary significantly depending on how it is elicited and how it is measured.

The fact that several genes resembling PRRs confer quantitative resistance in various plant species^29^ and that the strength of flg22 perception is negatively correlated with susceptibility to *Pseudomonas syringae*^22^ suggest some connection between variation in the MTI response and quantitative disease resistance. However, the relationship between these traits is not well understood, especially in crop plants as opposed to model systems such as Arabidopsis. The objectives of this study were to identify QTL for TLS resistance and the MAMP response and then to investigate the relationship between the MAMP response in sorghum and target leaf spot disease resistance.

## Results and Discussion

### Linkage map construction

A linkage map was generated for the BTx623/BTx642 RIL population using 1020 SNP markers. The map spanned a length of 4,689 cM or 632,560,609 Mb with 12 linkage groups representing the 10 chromosomes of sorghum and covering approximately 92% of the genome (File S1). Chromosome arms for 3 and 10 could not be joined into one linkage group resulting in linkage groups 3A, 3B, 10A, and 10B. The map contained an average marker coverage of one marker per 5.2 cM or 654,684 Mb (Table S2). A previously-available linkage map for the BTx623/SC155-14E RIL population was used (Table S2, File S1)^30^.

#### Field evaluation of TLS resistance

**Figure 1:**
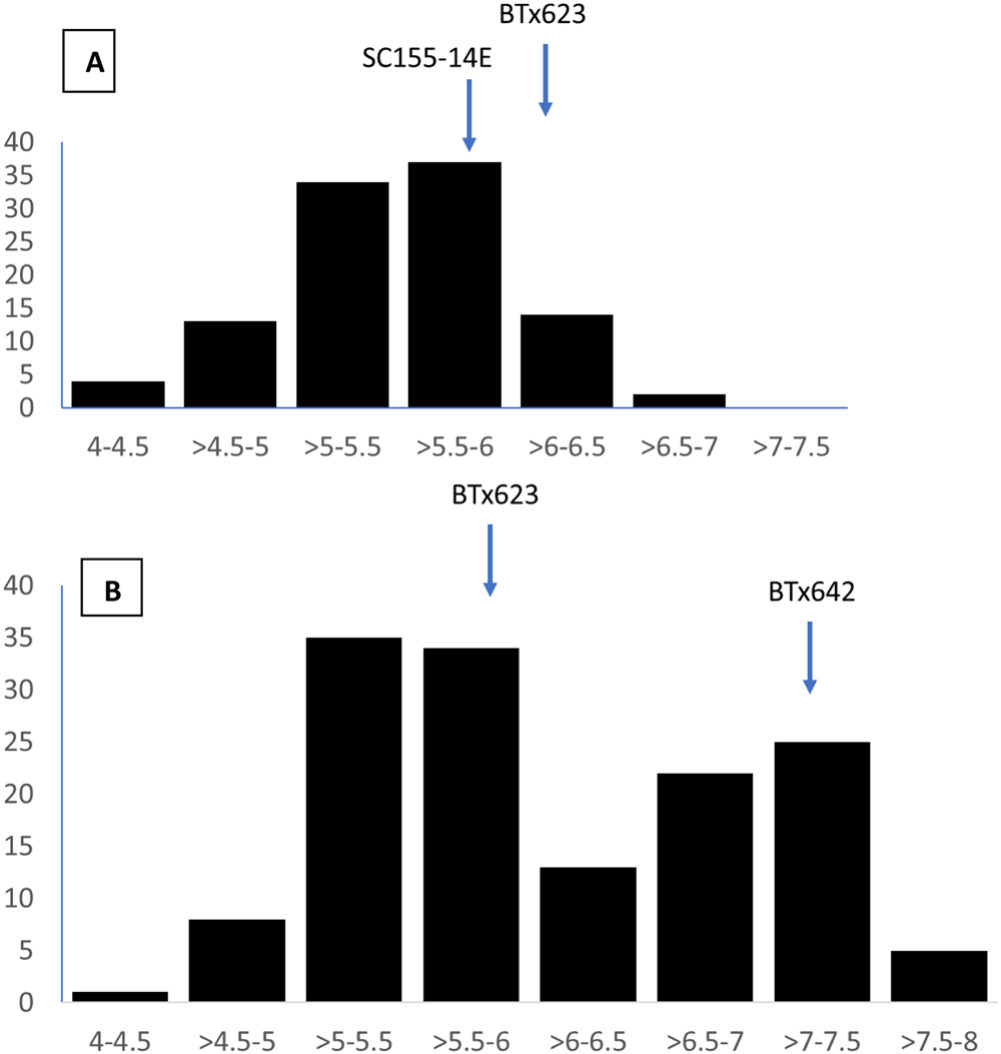
Distribution of TLS resistance LS mean scores in the (**A**) BTx623/SC155–14E and (**B**) BTx623/BTx642 populations. Parental values are indicated.

Two RIL populations, BTx623/SC155-14E and BTx623/BTx642, were selected for study based on variation of the parental lines for both TLS and MAMP response (see below). Both populations were assessed in 2016 and 2017 in randomized complete blocks with 2 full replications per year. For both populations, significant differences were identified for genotype, year of collection, replication for each experiment within each year, scoring date within each year as well as the genotype by replication and genotype by year interactions (Table 1). For the BTx623/SC155-14E RIL population, The TLS ratings of the parents, BTx623 and SC155-14E, were similar (Average of 6.07 and 5.77 respectively, Table 2 and Fig. 1). Nevertheless significant transgressive segregation was observed, RIL average values ranging from 4.1 to 6.8 (Table 2, Fig. 1) and the correlation between the average line scores for 2016 and 2017 was 0.38 (*P* < 0.0001, Table 3). For the BTx623/BTx642 RIL population, more divergent TLS scores were observed for the parents, with average scores 5.84 and 7.17 for BTx623 and BTx642 respectively (Table 2, Fig. 1). The range of scores for the RILs was likewise broader than for the BTx623/SC155-14E RIL population ranging on average from 4.38 to 7.75, again showing significant transgressive segregation. The correlation between the average line scores for 2016 and 2017 was 0.74 (*P* < 0.0001, Table 3).

**Table 1:** Analysis of variance significance values for BTx623/SC155-14E and BTx623/BTx642 populations evaluated in 2 replications over two years for target leaf spot severity as well as two individual runs for ROS response to flg22 treatment.

Traits	Source	DF	Mean Square	F value^†^
**BTx623/SC155-14E RIL population**				
Target Leaf Spot	Genotype	102	2.10	4.23***
	Year	1	28.73	57.75***
	Replication(Year)	2	158.38	318.41***
	Scoring Date(Year)	2	285.18	573.33***
	Genotype*Replication(Year)	202	0.77	2.03***
	Genotype*Year	103	1.01	1.54**
	Genotype*Date(Year)	206	0.42	0.84
flg22 response	Genotype	102	96,066,499	5.43***
	Run	1	151,043,235	8.53*
	Replication(Run)	14	6,803,643	0.38
	Genotype*Run	100	225,642,553	1.27*
	Genotype*Replication(Run)	1377	7,576,533	0.43
**BTx623/BTx642 RIL population**				
Target Leaf Spot	Genotype	148	5.74	15.30***
	Year	1	184.63	492.18***
	Replication(Year)	2	192.01	511.86***
	Scoring Date(Year)	2	274.89	732.79***
	Genotype*Replication(Year)	295	0.54	1.45**
	Genotype*Year	150	1.02	2.73***
	Genotype*Date(Year)	300	0.34	0.89
flg22 response	Genotype	148	102,595,497	15.04***
	Run	1	22,049,841	0.88
	Replication(Run)	14	81,392,900	1.91*
	Genotype*Run	150	310,017,289	4.75***
	Genotype*Replication(Run)	1958	51,492,577	0.78

^†,^***P < 0.0001; **P < 0.01; *P < 0.05.

**Table 2:** Adjusted means for parents, BTx623, SC155-14E, BTx642, and BTx623/SC155-14E and BTx623/BTx642 populations, minimum, and maximum trait values for TLS severity calculated for two individual field environments and an across environment calculation as well as ROS response to flg22 treatment for two individual runs and an across run calculation.

Experiment^†^	Target Leaf Spot Severity			flg22 (RLUs)		
	2016	2017	Across	Run 1	Run 2	Across
Experiment^†^	Target Leaf Spot Severity			flg22 (RLUs)		
	2016	2017	Across	Run 1	Run 2	Across
BTx623	6.00	6.13	6.07	1,121	486	803
SC155-14E	6.13	5.42	5.77	42,796	35,367	39,081
Population Min	3.38	3.75	4.10	0	0	244
Population Max	7.39	7.00	6.80	232,256	145,687	188,972
Population Avg	5.68	5.39	5.77	14,783	9,401	12,092
BTx623	6.44	5.25	5.84	745	1,130	937
BTx642	7.75	6.58	7.17	28,232	21,469	24,850
Population Min	4	4.25	4.38	0	0	278
Population Max	8.25	7.5	7.75	375,835	118,229	247,032
Population Avg	6.54	5.77	6.15	13,031	12,885	12,958

**Table 3:** Pearson correlation coefficients among different experimental runs of target leaf spot severity testing as well as total ROS production triggered by MAMPs in the BTx623/SC155-14E and BTx623/BTx642 RIL populations.

BTx623/SC155-14E RIL population					
	TLS 2016	TLS 2017	TLS average	flg22 run 1	flg22 run 2
	TLS 2016	TLS 2017	TLS average		flg22 run 2
TLS 2016	—				
TLS 2017	0.38***	—			
TLS average	0.86***	0.79***	—		
flg22 run 1	−0.08^NS^	−0.07^NS^	−0.10^NS^	—	
flg22 run 2	0.08^NS^	−0.01^NS^	−0.05^NS^	0.52***	—
flg22 average	−0.02^NS^	−0.06^NS^	−0.05^NS^	0.95***	0.83***
**BTx623/BTx642 RIL population**					
TLS 2016	—				
TLS 2017	0.74***	—			
TLS average	0.95***	0.91***	—		
flg22 run 1	−0.02^NS^	0.02^NS^	−0.00^NS^	—	
flg22 run 2	0.11^NS^	0.10^NS^	−0.11^NS^	0.31***	—
flg22 average	0.03^NS^	0.05^NS^	0.04^NS^	0.95***	0.84***

†***P < 0.0001; **P < 0.01; *P < 0.05; ^NS^non-significant.

### TLS QTL mapping

QTL for TLS severity were detected in both populations. QTL analysis was performed with phenotypic data from individual environments and also with line averages across environments (Table 4). Permutation testing estimated a genome-wide significance threshold of 2.5 for TLS scores.

**Table 4:** Characteristics of QTL associated with target leaf spot severity and flg22-induced ROS production for the RIL populations, BTx623/SC155-14E and BTx623/BTx642.

BTx623/SC155-14E RIL population								
Trait	Env	LG	Peak cM	Peak bp	LOD	Additive Effect	*R*^2^ (%)	Support Interval (cM)
Trait	Env	LG	Peak cM	Peak bp	LOD	Additive Effect	*R*^2^ (%)	Support Interval (cM)
TLS	2016	3	11.41	1,648,243	3.46	−0.25	11.64	6.6–21.2
TLS	2017	3	12.91	1,964,497	4.78	−0.27	15.54	11.4–17.9
TLS	**across**	**3**	**10.61**	**1,514,563**	**4.84**	**−0.22**	**14.88**	**5.3–18.6**
TLS	2017	4	103.41	61,654,830	3.27	0.22	11.49	101.2–106.6
TLS	**across**	**4**	**103.41**	**61,654,830**	**4.82**	**0.23**	**15.95**	**101.2–106.8**
flg22	run 2	4	19.41	2,164,505	2.92	−0.29	10.13	8.9–26.5
flg22	run 1	6	73.31	55,771,287	3.19	−0.22	11.28	72.8–81.5
**flg22**	**across**	**6**	**72.41**	**55,131,463**	**2.72**	**−0.22**	**10.16**	**65.2–73.3**
flg22	run 2	8	68.71	56,474,814	3.72	−0.24	17.35	64.7–70.4
**BTx623/BTx642 RIL population**								
TLS	2016	5	125.31	7,752,952	38.3	−0.93	65.65	124.2–126.4
TLS	2017	5	125.31	7,752,952	22.0	−0.51	43.99	123.3–129
TLS	**across**	**5**	**125.31**	**7,752,952**	**39.7**	**−0.74**	**71.12**	**123.8–126.8**
TLS	2016	6	49.01	50,702,998	3.2	0.19	3.08	42.6–56
TLS	2017	9	315.81	39,951,155	2.6	0.22	3.92	315.5–317.9
TLS	2017	9	329.31	45,761,129	5.2	0.39	8.34	324.3–334.5
TLS	2017	9	346.41	46,375,421	2.8	−0.27	4.39	342.9–354.7
**TLS**	**across**	**9**	**316.81**	**40,059,782**	**8.3**	**0.45**	**8.87**	**315.7–320.4**
flg22	run 1	4	139.21	12441343	3.15	0.24	7.39	138.3–148.8

In the BTx623/SC155-14E RIL population a TLS resistance QTL was detected on chromosome (chr.) 3 at around 10.6 cM in both environments and across environments. An additional TLS QTL on chr. 4 at 103.4 cM was detected in the 2017 and across environments (Table 4). The SC155-14E and BTx623 contributed the resistance alleles on chromosomes 3 and 4 respectively, which does provide explanation of the transgressive variation observed in this population.

In the BTx623/BTx642 RIL population, a major TLS QTL on chr. 5 was detected in both 2016 and 2017 using average values across environments, explaining 71.1% of the variation across environments (Table 4). Additional QTL were identified; one on chr. 9 was identified in 2017 across environments and one on chr. 6 was detected in 2016. For the QTL detected across environments, BTx642 contributed the resistance allele on chr. 5 and BTx623 contributed the resistance allele on chr.9.

Considering just the TLS QTL detected across environments, the chr. 5 QTL detected in the BTx623/BTx642 RIL population maps precisely to the position of the major TLS resistance gene *ds1*^3^. Combined with its large effect the fact that it accounts for a large majority of the variation observed, this suggests that BTx642 carries the recessive ds1 resistance gene and that BTx623 does not. Since no QTL is detected at this locus in the BTx623/SC155-14E population, this also implies that SC155-14E also carries the dominant susceptibility allele at this locus. A TLS QTL was detected on chr. 3 in a previous study^5^ but this was not close to the QTL mapped here.

### MAMP response evaluation

**Figure 2:**
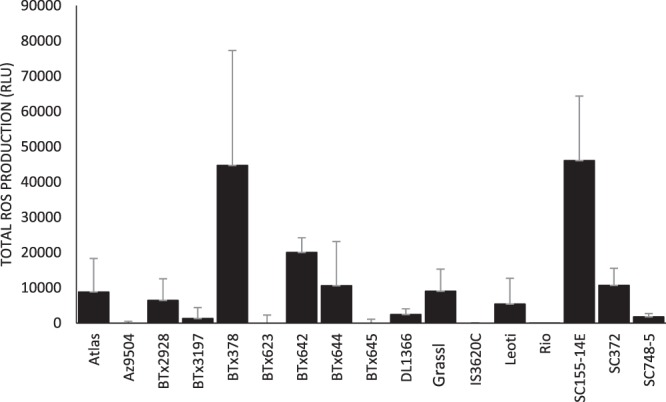
flg22-induced ROS production in various sorghum lines.

To initially assess variation within available germplasm, ROS production of sixteen sorghum parental lines (Table S1) was assessed in response to two MAMP treatments, flg22 and chitin. Variable responses between lines were identified for both flg22 and chitin treatments however, chitin responses were inconsistent between replications, so only results of the flg22 response are reported here (Fig. 2). It is not clear why the chitin response data were inconsistent between replications since we had achieved relatively consistent results using a very similar assay in maize^23^. While every effort was made to standardize conditions between replications, it is possible that there were sources of variation that were not accounted for. Lines BTx378, BTx642, and SC155-14E, and SC372 were the highest responders to flg22 and lines Az9594, BTx3197, BTx623, DL1366, Rio, and SC748-5 were among the lowest responders (Fig. 2).

**Figure 3:**
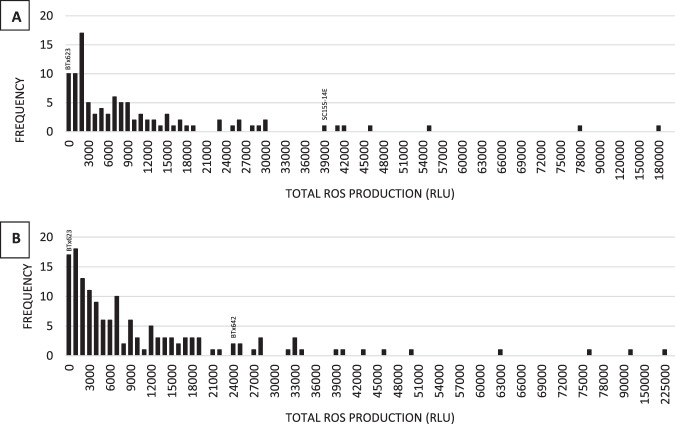
Distribution of total ROS production (RLU) in response to flg22 in (**A**) BTx623/SC155–14E RIL population and (**B**) BTx623/BTx642 RIL population.

We assessed both sorghum RIL populations for flg22-induced ROS production response. Substantial variation in levels of ROS production was observed for both RIL populations (Fig. 3). Significant effects were identified for genotype, run, and the interaction between genotype and run in both populations (Table 1). The correlations between runs were 0.52 and 0.31 for the BTx623/SC155-14E and BTx623/BTx642 populations respectively (both *P* < 0.0001). Other parameters for the flg22 response in the two population are shown in Table 1. For the average flg22 response values, one QTL on chr. 6 was identified In the BTx623/SC155-14E RIL population and none in the BTx623/BTx642 population (Table 4). Permutation testing estimated a genome-wide significance threshold of 2.3 for flg22 response scores.

#### Comparison of TLS and MAMP response data

There was no significant correlation between TLS resistance scores and flg22-induced ROS response scores in either population (Table 3), nor was there any correspondence between QTL identified for the two traits. It seems therefore that the loci and genes controlling variation in these two traits are entirely distinct. Arguably this is not surprising as flg22 is a bacterial MAMP while TLS is caused by a fungus. It was unfortunate in this respect that we were not able to detect a consistent response to chitin. As discussed in the introduction, Zhang *et al*.^23^ found a strong correlation between responses to flg22 and chitin in a maize mapping population. Nevertheless, no correspondence to response to fungal disease was detected in this previous study either.

Also as discussed above, the MAMP response can be measured in a number of different ways and these different methods give substantially different results^23,27^. It is therefore premature to say that variation in the strength of the MAMP response did not affect levels of disease resistance in our experiments. However we were not able to generate any evidence to support this hypothesis.

In conclusion, this study detected several previously unidentified QTL for TLS resistance. It also reports variation between sorghum lines in the flg22-induced ROS response and identifies one QTL associated with variation in this response. No evidence was found to link variation in the MAMP response to variation in TLS resistance

## Materials and Methods

### Plant materials

All sorghum lines were provided by Dr. William Rooney (Texas A&M University,) and Dr. Stephen Kresovich (Clemson University). These consisted of 17 diverse lines that were parents of several mapping populations and two recombinant Inbred Line (RIL) populations (Table S1). The two RIL populations were both developed at Texas A&M and were at the F_5:6_ stage. BTx623 – the standard sorghum line used for genetics^31^- was a parent of both populations, the other parents being BTx642 and SC155-14E. BTx623, is a TLS susceptible elite line^3^, SC155-14E, is a line developed for anthracnose resistance^30^ and BTx642, is a derivative of the stay-green conversion line SC35 developed by Rosenow *et al*.^32^. The BTx623/SC155-14E and BTx623/BTx642 populations consisted of 103 and 149 lines respectively.

### MAMP Response Testing

Two MAMPs, flg22 and chitin, were used in this study. Flg22 is a peptide corresponding to a conserved domain of bacterial flagellin and was ordered from Genscript (catalog# RP19986). The 20 X chitin mixture is made by adding 20 mg of chitin powder (Sigma-Aldrich, catalog# C3641) to 10 ml ddH_2_O, vortexing for 30 s, letting settle for 10 min, vortexing again for 30 sec and letting settle for 10 min Any undissolved chitin settled to the bottom of the tube and the clear supernatant above was used for the experiment.

ROS assays were carried out according to Zhang *et al*.^23^ with modification. One leaf disc (diameter 5.5 mm) from four 15 day-old sorghum plants of each genotype were excised from and floated on 50 µl ddH_2_0 in a white 96-well plate (Fisher Scientific, catalog# 353296) overnight in dark. Immediately before testing, 50 µl of 2X reaction solution carrying 1 µl of 5mM L-012 (a chemiluminescence probe that reacts with superoxide anions and produces luminescence at long wavelengths; ordered from Wako Pure Chemical Industries, Ltd. catalog# 120-04891) in ddH_2_O, 1 µl of 2 mg/ml horseradish peroxidase (Sigma-Aldrich catalog# P6782), and 1 µl of 100 µM flg22 or 5 µl of the chitin mixture, was added to the 96-well plate. Immediately after treatment, the chemiluminescent signal from each well was recorded for 30 min using a Photek CCD camera 216 (Photek ltd., East Sussex, U.K).

To assess the variation of the MAMP response within individual genotypes, each genotype was assessed in sixteen wells of each 96-well plate. Eight wells consisted of a mock treatment (without MAMPs) and eight wells consisted of treatment (with MAMPs). The parents of the populations, BTx623, SC155-14E, and BTx642, were always included as repeated checks on each 96-well plate while we test the ROS product of the RILs populations. Eight biological replicates of responses to flg22 and chitin mixture were measured in every case with genotypes re-randomized in each of the two runs.

#### Inoculum preparation and inoculation procedure

Inoculum was prepared essentially as described previously^33^. Sorghum grain was soaked from 3 to 4 days in water, placed in 1 L flasks, and autoclaved for one hour (120 PSI and 121 °C). Autoclaved grain was inoculated with *Bipolaris cookei* isolate LSLP18 received from Dr. Burt Bluhm (University of Arkansas). The fungus grew at room temperature (23–25 °C) for approximately 14 days until the sorghum was colonized with the fungus. The sorghum was air-dried and stored at 4 °C. The dried infested sorghum was used to inoculate the entire trial. Sorghum plants were inoculated 30–40 days after planting by adding 6 to 10 infested sorghum kernels placed into the whorl of each plant.

### Field evaluation of target leaf Spot resistance

RIL populations along with parental checks were planted at the Central Crops Research Station in Clayton, NC on May 15 2016 and June 14–15 2017 in a randomized complete block design with two experimental replications. Experiments were planted in 1.8 m single rows with a 0.9 m row width using 10 seeds per plot. Two border rows were planted around the periphery of each experiment to prevent edge effects. Rows were inoculated on July 1 2016 and July 15 2017 of each plant at sorghum growth stage 3. Visual disease ratings were taken two times at ~14-day intervals starting three weeks after inoculation. Ratings were taken visually on a one to nine percentage scale with 1 = a dead plant; 2 = > 90% of leaf area showing lesions; 3 = > 75%; 4 = > 60%; 5 = > 45%; 6 = > 30%; 7 = > 15%; 8 = > 5%; and 9 = no visible symptoms on leaves.

### Phenotypic data analysis

Data was analyzed with SAS software v9.4^34^. An analysis of variance (ANOVA) and least square (LS) means were generated using the GLM procedure for target leaf spot severity and ROS response to flg22 treatment. Mock treatment values for each line were subtracted from the mean of each line response before analysis as a normalization technique. Standard F-tests in all analyses were used to determine significance of main effects and interactions. In the model, replications and environments were considered random, while the main effects were considered fixed. Individual hypothesis testing of effects and interactions was designated using the appropriate error term in the GLM procedure. Pearson correlation coefficients were calculated for TLS severity and ROS response to flg22 and chitin treatments using the CORR procedure in SAS.

### Genotypic analysis and linkage mapping

Genotypic data and a genetic linkage map for the BTx623/SC155-14E RIL population was provided by Dr. Patricia Klein in the Department of Horticultural Sciences and Institute for Plant Genomics and Biotechnology at Texas A&M University, College Station, TX and has been previously published^30^. Genotypic data for the BTx623/BTx642 RIL population were generated using Illumina sequencing of reduced-representation of genomic libraries as described previously^35^. Briefly, DNA was extracted using a modified CTAB protocol, quantified using PicoGreen, double-digested with *Pst*I-HF and *Hin*P1I, and ligated to barcoded adapters. Individual libraries were then pooled, bead-cleaned, PCR amplified, and bead-cleaned again. Average size and concentration of pooled libraries were estimated using a DNA7500 chip on an Agilent Bioanalyzer, and libraries were sequenced on an Illumina HiSeq. 2000 instrument. SNP calling was performed using the TASSEL5 GBS pipeline^36^ and missing data were imputed using Beagle4 (Browning and Browning 2007). To create the linkage map for the BTx623/BTx642 population a χ² test was performed for each marker to test for segregation distortion. Markers showing the appropriate Mendelian segregation ratios for individual markers were used to construct a genetic linkage map using Joinmap® 4.0 software^37^. Linkage groups were assigned using the LOD score of 5.0. Map distances were calculated using the Kosambi mapping function^38^. Summary statistics for each map are shown in Table S2 and the actual map positions of each marker are provided in File S1.

### QTL Mapping

The composite interval mapping (CIM) option in Windows QTL cartographer 2.5^39^ was used to identify QTL, estimate LOD scores, and measure the proportion of phenotypic variation explained by individual QTL for individual environments as well as across environments. Default settings for CIM were used with the standard model, a control marker number set to 5, a window-size of 10, and a walk speed (cM) of 2. A permutation test with 500 iterations was conducted to establish a *P* < 0.05 threshold for the detection of putative QTL (Churchill and Doerge 1994).

## Supplementary information

Supplementary Tables S1 and S2

Supplementary File S1
