# Supplementary material for: Identification of QTL for Target Leaf Spot resistance in Sorghum bicolor and investigation of relationships between disease resistance and variation in the MAMP response
Source: Sci Rep. 2019 Dec 4;9:18285. doi: 10.1038/s41598-019-54802-x (PMC6893015; doi:10.1038/s41598-019-54802-x)
Supplement: Supplementary file 1 — Supplementary Tables S1 and S2 [file 41598_2019_54802_MOESM1_ESM.pdf]

Supplemental Tables for:

Identification of QTL for Target Leaf Spot resistance in *Sorghum bicolor* and investigation of relationships between disease resistance and variation in the MAMP response.

Jennifer Kimball[1], Yaya Cui[2], Dongqin Chen[2], Pat Brown[3], William Rooney[4], Gary Stacey[2], and Peter Balint-Kurti[5,6,*]

[1,5] Department of Agronomy and Plant Genetics, University of Minnesota, St. Paul, MN 55108, USA

[2]Divisions of Plant Science and Biochemistry, C. S. Bond Life Science Center, University of Missouri, Columbia, MO 65211, USA

[3]Department of Plant Sciences, University of California Davis, Davis CA 95616, USA

[4]Department of Soil and Crop Sciences, Texas A&M University, College Station, TX 77843, USA

[5]Dept of Entomology and Plant Pathology, NC State University, Raleigh NC 27695, USA

[6]Plant Science Research Unit, USDA-ARS, Raleigh NC 27695, USA

[*]Author for Correspondence

Table S1. Germplasm list of sorghum lines and RIL populations utilized in this study.

Line	# lines	Source
Atlas	1	Kresovich
Az9504	1	Rooney
BTx2928	1	Rooney
BTx3197	1	Rooney
BTx378	1	Rooney
BTx623	1	Rooney
BTx642	1	Rooney
BTx644	1	Rooney
BTx645	1	Rooney
DL1366	1	Rooney
Grassl	1	Kresovich
IS3620C	1	Rooney
Leoti	1	Kresovich
Rio	1	Kresovich
SC155-14E	1	Rooney
SC372	1	Rooney
SC748-5	1	Rooney
BTx623/SC155-14E RIL population	103	Rooney
BTx623/BTx642 RIL population	149	Rooney

Table S2. SNP marker distribution and coverage over 10 linkage groups identified in two sorghum RIL population, BTx623/SC155-14E and BTx623/BTx642.

RIL Population	Linkage Group	Total Markers	Coverage (cM)	Coverage (Mb)	Average Marker Coverage (cM)	Average Marker Coverage (Mb)
**BTx623/SC155-14E**	1	166	183.24	80,072,613	1.11	485,289
	2	114	163.71	77,183,847	1.45	683,044
	3	130	129.13	73,013,986	0.99	561,646
	4	109	137.84	68,020,841	1.28	629,823
	5	81	114.85	71,427,155	1.42	881,816
	6	96	97.68	60,245,023	1.03	634,157
	7	66	103.28	63,234,526	1.56	958,098
	8	60	113.51	61,843,660	1.92	1,048,199
	9	42	71.24	49,608,749	1.74	1,209,968
	10	80	108.24	60,258,011	1.23	684,749
	Total	952	1,222.72	664,908,411	-	-
**BTx623/BTx642**	1	88	476.051	80,060,912	5.41	909,783
	2	148	691.118	76,486,630	4.67	516,802
	3A	32	134.283	7,001,807	4.20	218,806
	3B	135	633.251	59,086,246	4.69	437,676
	4	51	235.268	56,824,762	4.61	1,114,211
	5	95	513.792	71,711,852	5.41	754,862
	6	55	257.631	59,160,437	4.68	1,075,644
	7	62	272.436	54,424,806	4.39	877,819
	8	138	528.288	61,861,454	3.83	448,271
	9	145	438.739	49,614,711	3.03	342,170
	10A	30	153.84	5,045,669	5.13	168,189
	10B	52	633.251	51,281,323	12.18	986,179
	Total	1031	4,968	632,560,609	-	-
